# Hypoxia inducible factor-1 alpha as a therapeutic target in multiple myeloma

**DOI:** 10.18632/oncotarget.1736

**Published:** 2014-01-23

**Authors:** Enrica Borsi, Giulia Perrone, Carolina Terragna, Marina Martello, Angela F. Dico, Giancarlo Solaini, Alessandra Baracca, Gianluca Sgarbi, Gianandrea Pasquinelli, Sabrina Valente, Elena Zamagni, Paola Tacchetti, Giovanni Martinelli, Michele Cavo

**Affiliations:** ^1^ Department of Experimental Diagnostic and Specialty Medicine (DIMES), “L. & A. Seràgnoli”, Bologna University School of Medicine, S. Orsola's University Hospital, Italy; ^2^ Fondazione IRCCS Istituto Nazionale dei Tumori, Hematology Department, Via Venezian 1, Milano, Italy; ^3^ Department of Biomedical and Neuromotor Sciences, University of Bologna, Via Irnerio 48, Bologna, Italy

**Keywords:** HIF-1α, Multiple Myeloma, Cell cycle, Mitochondria, Warburg effect

## Abstract

The increasing importance of hypoxia-inducible factor-1α (HIF-1α) in tumorigenesis raises the possibility that agents which specifically inhibit this transcription factor, would provide significant therapeutic benefit. The constitutive expression of HIF-1α in about 35% of Multiple Myeloma (MM) patients suggests HIF-1α suppression might be part of a therapeutic strategy. Accordingly, we explored the effect of EZN-2968, a small 3^rd^ generation antisense oligonucleotide against HIF-1α, in a panel of MM cell lines and primary patients samples. Here, we demonstrated that EZN-2968 is highly specific for HIF-1α mRNA and that exposure of MM cells to EZN-2968 resulted in an efficient and homogeneous loading of the cells showing a long lasting low HIF-1α protein level. In MM cells, HIF-1α suppression induced a permanent cell cycle arrest by prolonging S-phase through cyclin A modulation and in addition it induced a mild apoptotic cell death. Moreover, HIF-1α suppression caused a metabolic shift that leaded to increased production of ATP by oxidative phosphorylation (i.e. Warburg effect reversion), that was confirmed by the observed mitochondrial membrane potential decrease. These results show that HIF-1α is an important player in MM homeostasis and that its inhibition by small antisense oligonucleotides provides a rationale for novel therapeutic strategy to improving MM treatment.

## INTRODUCTION

The hypoxia inducible factor family members (HIFs) are crucial effectors in either normal cell homeostasis or tumorigenesis. HIF-1α has been recognized as the most important player in hypoxia response and it is widely implicated in tumor survival and progression [1-2]. HIF proteins are heterodimers of one of three major oxygen sensitive HIF-α subunits (HIF-1α, HIF-2α or HIF-3α) associated with a constitutive HIF-1β subunit (also known as Aryl hydrocarbon Receptor Nuclear Translocator or ARNT), which together form the HIF-1, HIF-2 and HIF-3 transcriptional complexes, respectively [3]. HIF-1α stabilization, is induced at low oxygen tension (hypoxia) and protein levels are regulated by post-translational modulation: under normoxic condition (oxygen tension higher than 3-5% according to cell type), the oxygen-dependent degradation (ODD) domain is hydroxylated and ubiquitinated for subsequent proteasomal degradation [4]. The transcriptional network of HIF-1α serves as a master controller of cellular response to hypoxia and regulates genes required for adaptation affecting cellular growth, metabolism and the angiogenic response [5].

In many cancer cells, abnormal expression of HIF-1α has been described [6-11] and associated with induction of pro-survival signalling pathways (such as MAPK, PI3K cascades) and c-MYC oncogene, as well as the metabolic shift known as Warburg effect [12-15]. Moreover, HIF-1α activates transcription of genes that play key roles in critical aspects of cancer biology, including stem cell maintenance (NOTCH1, WNT-β catenin pathways) [16], angiogenesis (VEGF, iNOS signalling) [17] and metastasis [18].

In solid tumors, increased HIF-1α protein expression has been related to clinical outcome and associated with higher risk of progression and mortality [7, 10, 19]. Preclinical data have suggested that HIF-1α suppression can result in anticancer effects in several xenograft tumor models [20, 21] and delay of the initial growth in primary tumors and metastases as shown in a mouse model of breast cancer [22]. Validation of HIF-1 as a therapeutic target in solid tumors has been suggested by both preclinical models and early clinical trials aimed to evaluate compounds interfering with HIF-1 gene regulation either indirectly (DNA intercalators, topoisomerase I and mTOR inhibitors) or directly (HIF selective inhibitors) [23]. While an acceptable therapeutic window of tolerability has been described, the anticancer role of HIF inhibition has not been established, and the precise mechanistic consequences of HIF inhibition remain elusive and may differ in various tumor types.

In Multiple Myeloma (MM), HIF-1α expression has been described in several cell lines and in about 35% of CD138^+^ cells isolated from MM patients samples [24], suggesting an oxygen independent stabilization of the protein [25]. *In vitro* studies have shown that several signal transducers induced by pro-survival cytokines (such as STAT3 and MAPK) positively regulate HIF-1α expression [26]; in addition, deregulation of c-MYC has been associated with HIF-1α up-regulation [27]. Finally, in MM cells HIF-1α regulates bone marrow angiogenesis through the stimulation of vascular endothelial growth factor (VEGF) [28, 29]. In preclinical studies, the inhibition of HIF-1α has been shown to enhance the sensitivity to melphalan [30] and early down-regulation of HIF-1α expression has been reported in MM cell lines sensitive to bortezomib and lenalidomide [24]. Recently TH-302, an hypoxia-activated “prodrug”, was shown to induce apoptosis in MM cell lines *in vitro* and in a xenograft mouse model [31]. Moreover, Storti *et al* [32] have also shown that HIF-1α down-regulation by shRNA produces significant tumor growth inhibition in JJN3 MM cell xenograft mouse model, associated with inhibition of angiogenesis and bone destruction. Based on these data, MM appears to be a good model for evaluating the mechanism and the biological role of HIF-1α inhibition.

Preliminary data of an ongoing phase 1 trial in advanced solid tumors testing the small antisense oligonucleotide EZN-2968, have shown a down-modulation of the target in skin biopsies of some patients and a favorable safety profile in patients with advanced solid tumors [33, 34]. In order to clarify the EZN-2968 mechanism of action, in this study, we evaluated the activity of EZN-2968 against HIF-1α in MM cells. We showed that HIF-1α mRNA and protein are constitutively present in MM cells and are further inducible by bone marrow milieu stimuli (such as IL-6 and IGF-1) even in normoxic culture conditions. In addition, we analyze the effect of the EZN cellular treatment on the HIF-1α expression level, the relationship between HIF-1α suppression and MM cells viability, apoptotic death, cell cycle and the Warburg-phenotype.

Our data strongly support the hypothesis that HIF-1α is an important actor in MM homeostasis and that its inhibition may suppress tumor growth by preventing proliferation of plasmacells through a delay in S-phase progression, possibly mediated by the switch towards a mitochondrial oxidative metabolism.

## RESULTS

### Constitutional and inducible expression of HIF-1α in human MM cells

**Figure 1:**
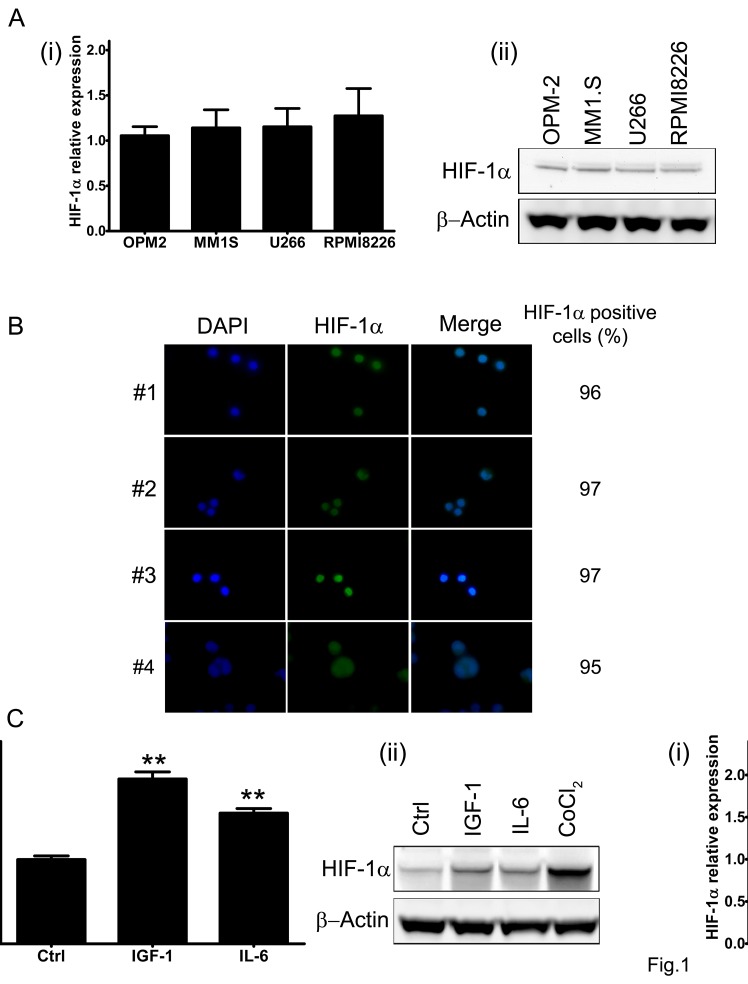
HIF-1α expression in multiple myeloma cells (A) Baseline level of HIF-1α mRNA (i) and protein product (ii) were assessed in four MM cell lines by qRT-PCR and Western Blotting (50 μg/lane) analysis, respectively. (B) HIF-1α protein expression in CD138^+^ cells derived from bone marrow aspirates from MM patients was monitored using immunofluorescence microscopy. The percentage of HIF-1α positive cells was calculated evaluating at least 200 cells. Green fluorescence: HIF-1α; Blue fluorescence: DAPI. (C) To evaluate the pro-survival stimuli modulation of HIF-1α mRNA expression (i) and protein synthesis (ii), the MM1.S cells were treated with IGF-1 (100 ng/ml) or IL-6 (50 ng/ml) for 4h. The effect of 24h treatment with CoCl_2_ (100 μM) on HIF-1α stabilization was taken as control. The immunoblot assays were performed twice with similar results. Representative data from one experiment are presented. mRNA expression of HIF-1α was normalized to GAPDH level. Histograms show the mean value ± SD of three independent experiments. ** p<0.01 compared to control.

We first investigated the baseline expression of HIF-1α in four human derived MM cell lines (MM1.S, RPMI8226, U266 and OPM-2). As shown in Fig. 1A (i) and (ii), HIF-1α mRNA and protein level were detectable in all MM cell lines tested under normoxic culture conditions (pO_2_ 21%). Furthermore, immunofluorescence analysis of CD138^+^ cells isolated from bone marrow aspirates from newly diagnosed MM patients, confirmed that HIF-1α protein is expressed in MM plasmacells (Fig. 1B). We next assessed whether pro-survival stimuli were able to modify HIF-1α expression. As summarized in Fig. 1C (i) and (ii) a marked up-regulation of HIF-1α expression, at both transcriptional (+50%) and protein level (+50%), was observed in MM1.S cells after short incubation with IGF-1 (100 ng/ml for 4h) whereas a moderate increase of HIF-1α mRNA (+35%) and protein level (+15%) was found when cells were treated with IL-6 (50 ng/ml), confirming that HIF-1α can be differentially induced by biological stimuli. As a positive control, MM1.S cells were treated with hypoxia-mimicking CoCl_2_ (100 μM for 24h), which is known to increase baseline protein levels of HIF-1α.

### EZN-2968 efficiently inhibits HIF-1α expression

**Figure 2:**
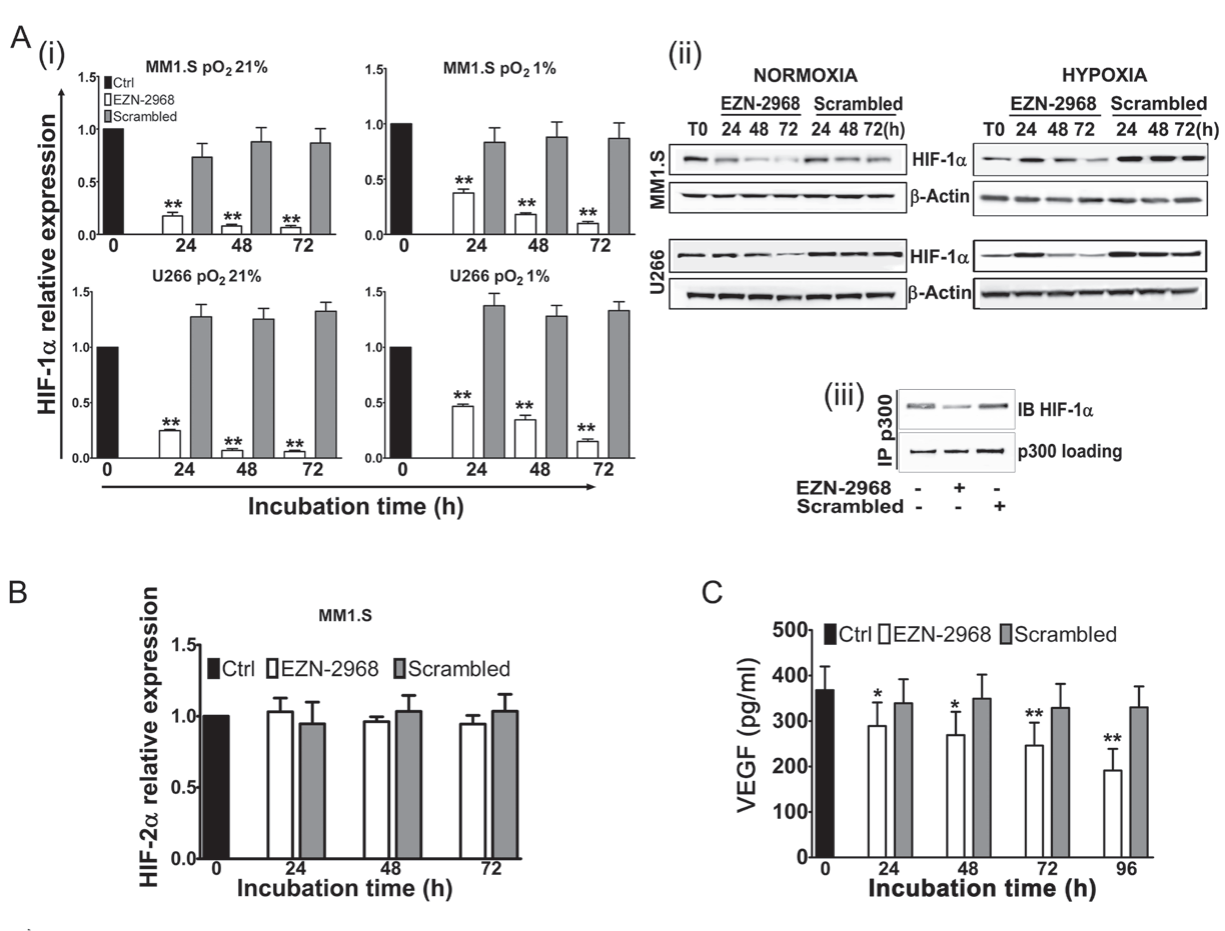
Reduction of HIF-1α levels induced by EZN-2968 oligonucleotide (A) MM1.S and U266 cell lines were incubated in the presence of either EZN-2968 or scrambled oligonucleotide (20 μmol/L) for up to 72h, in normoxic (pO_2_ 21%) or hypoxic (pO_2_ 1%) culture conditions. (i) HIF-1α mRNA expression (normalized to GAPDH level) and (ii) protein level (β-actin used as loading control) were then analyzed by qRT-PCR and Western Blotting, respectively. (iii) Immunoprecipitation assay was performed using anti-p300 antibody followed by immunoblotting assay using anti-HIF-1α antibody. MM1.S cells were treated with the HIF inhibitor for 48h prior to the assay. Representative data from one experiment are presented. (B) MM1.S cells were incubated in the presence of either EZN-2968 or scrambled oligonucleotide (20 μmol/L) up to 72h, and mRNA expression of HIF-2α was evaluated by qRT-PCR. (C) VEGF protein level secreted by MM1.S cells exposed to either EZN-2968 or scrambled oligonucleotide (20 μmol/L) were quantified by ELISA. Histograms show the mean value ± SD of three independent experiments. * p<0.05 and ** p<0.01 compared to control. EZN-2968-*white bars*, Scrambled-*grey bars*, Control-*black bars*.

To assess the specificity of the compound toward HIF-1α mRNA, MM cell lines (MM1.S and U266) were cultured under normoxia (pO_2_ 21%), with either EZN-2968 or scrambled oligonucleotide (20 μmol/L) for up to 72h. As shown in Fig. 2A a long lasting and time dependent inhibition of both HIF-1α transcription (i) and protein level (ii) was observed in EZN-treated samples compared to controls. Moreover, in order to demonstrate the efficiency of EZN-2968 delivery into MM cells, we perform immunofluorescence analysis using MM1.S cells. Notably, EZN-2968 was able to induce a potent down-regulation of HIF-1α protein levels in a time dependent manner. Remarkably, EZN-2968 was able to deliver into MM cells homogeneously without using any transfection methods (Supplementary S1). No significant change of HIF-1α expression was observed in scrambled oligonucleotide treated MM cells compared to controls. Incidentally, a down-modulation of HIF-1 expression, at both transcriptional and protein level was also detected in hypoxic culture conditions (pO_2_ 1%) (Fig. 2A (i) and (ii)).

To confirm the inhibition of HIF-1α activity, we performed a co-immunoprecipitation assay using anti-p300 antibody, and we showed that the interaction between p300, a nuclear coactivator of HIF-1α, and HIF-1 was reduced in EZN-2968-treated samples compared to untreated samples after 48h, suggesting a reduced HIF-1 transcriptional activity (Fig. 2A (iii)).

Owing to the homology in the sequence of the hypoxia inducible transcription factors, we investigated whether EZN-2968 was able to influence HIF-2α expression. As shown in Fig. 2B, in MM1.S cells, HIF-2α mRNA expression was not affected by the EZN-2968 treatment. Similar results were also obtained in U266, RPMI8226 and OPM-2 cell lines (data not shown). Finally, we tested whether the vascular endothelial growth factor (VEGF), a downstream target of HIF-1α transcriptional activity, was modulated by EZN-2968. MM1.S cells were grown in the presence of either EZN-2968 or scrambled oligonucleotide (20 μmol/L) up to 96h. The media were then collected and VEGF production was measured by ELISA. We found that the amount of VEGF in the media was reduced in a time dependent manner in the EZN-2968 treated MM cells compared to control, showing a reduction of about 50% when MM cells were exposed to EZN-2968 for 96h, supporting the direct involvement of HIF-1 in the control of VEGF release (Fig. 2C). Moreover, this observation was also confirmed in immunofluorescence analysis of MM1.S cells treated with EZN-2968 (data not shown).

### Effect of EZN-2968 on cell growth of MM cell lines and primary cells

**Figure 3:**
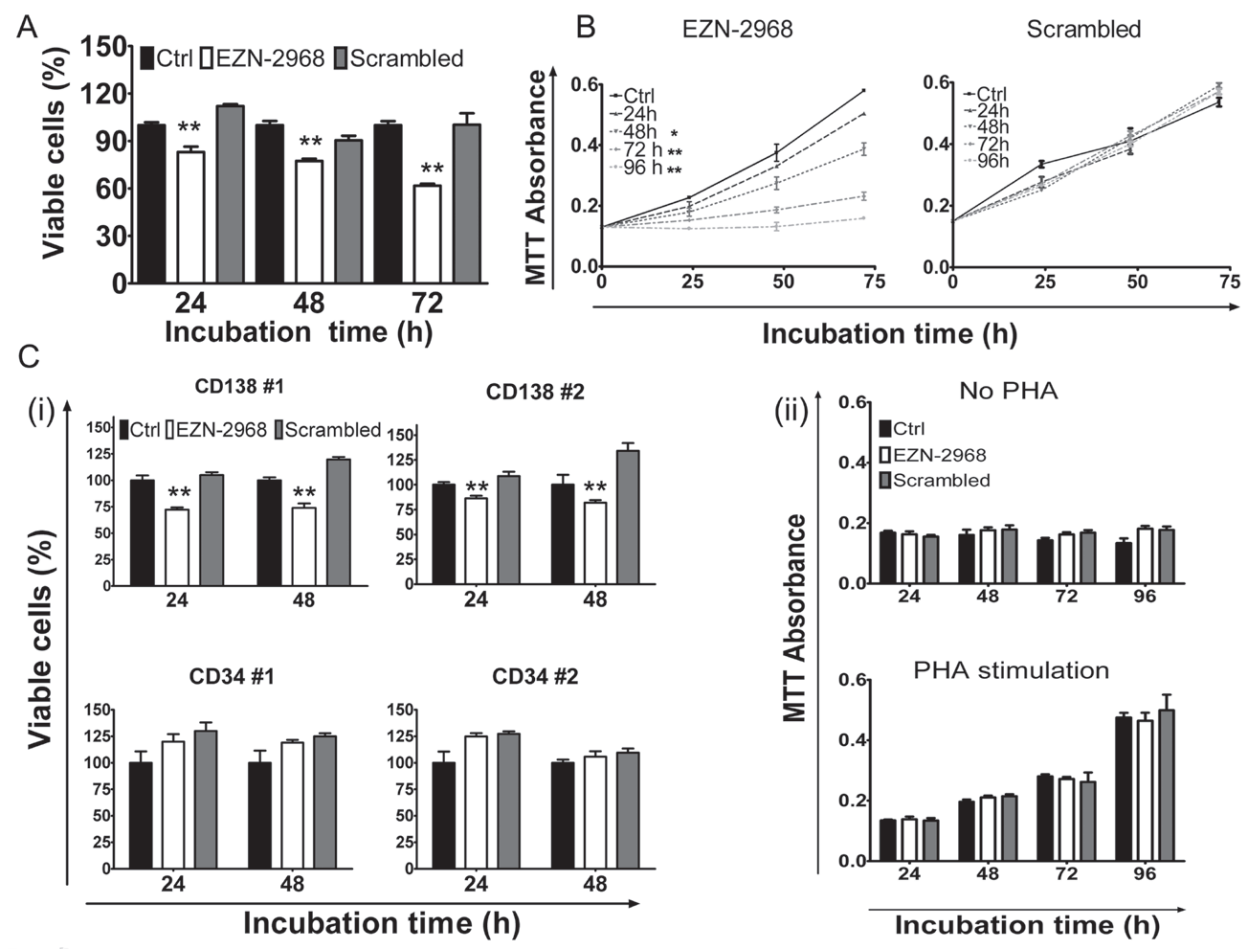
Viability of MM cells (A) The viability of MM1.S cells exposed to either EZN-2968 or scrambled oligonucleotide (20 μmol/L) was evaluated at 24h, 48h and 72h by MTT colorimetric survival assay. (B) Cell death commitment assay was performed to evaluate the irreversible impact on cell viability of EZN-2968. MM1.S cells were exposed to EZN-2968 (20 μmol/L) for up to 96h followed by drug washout and further incubation in drug free medium for additional 3 days. (C) (i) CD138^+^ cells from MM patients and CD34^+^ cells from healthy donors were purified and grown with either EZN-2968 or scrambled oligonucleotide (20 μmol/L) for 24h and 48h. Viable cells were measured by the MTT colorimetric survival assay. (ii) PBMCs isolated from healthy donors were incubated with either EZN-2968 or scrambled oligonucleotide (20 μmol/L) for up to 96h in the presence or absence of *phytohemagglutinin* stimulation (1 μg/μl). Values shown in histograms are mean ± SD of three independent experiments. * p<0.05 and ** p<0.01 compared to control. EZN-2968-*white bars*, Scrambled-*grey bars*, Control-*black bars*.

Under normoxia, the viability of MM1.S cell line at 24h, 48h, and 72h was 80%, 77% and 62%, respectively in the presence of EZN-2968 compared to untreated cells (Fig. 3A). The viability of MM1.S cell line was similarly reduced by EZN-2968 treatment under hypoxia, as compared to normoxic condition (Supplementary S2B). A similar effect was shown with U266 cell line, whereas under hypoxia the OPM-2 and RPMI8226 cells showed a lower sensitivity to EZN-2968 treatment, and the effect on cell viability was only observed at 72h (Supplementary S2A and S2B). Subsequently, MM cells were exposed to either EZN-2968 or scrambled oligonucleotide for up to 96h, followed by drug washout, and then grown in drug-free medium for 72h. In drug-free medium, MM cells showed growth inversely correlated with the time of previous exposure to the HIF inhibitor (Fig. 3B and Supplementary S3). In particular, we observed that cell proliferation was irreversibly affected by exposure to EZN-2968 as short as 48h. To validate the results obtained on MM cell lines, we next evaluated the effect of EZN-2968 on primary cells from MM patients. As summarized in Fig. 3C, selective inhibition of HIF-1α resulted in a similar reduction of cell viability at 24h and 48h, showing a decrease of about 14% and 28% in CD138^+^-treated cells compared to the control, respectively. Notably, CD34^+^ cells and peripheral blood mononuclear cells (PBMCs) derived from healthy donors cultured in the same experimental conditions were not affected by the treatment (Fig. 3C (i) and (ii)), even when the PBMCs were stimulated to proliferate non-specifically with *phytohemagglutinin* (PHA). Therefore the viability of non malignant cells was barely sensitive to EZN-2968.

### Ultrastructural study of MM cells after EZN-2968 exposure

**Figure 4:**
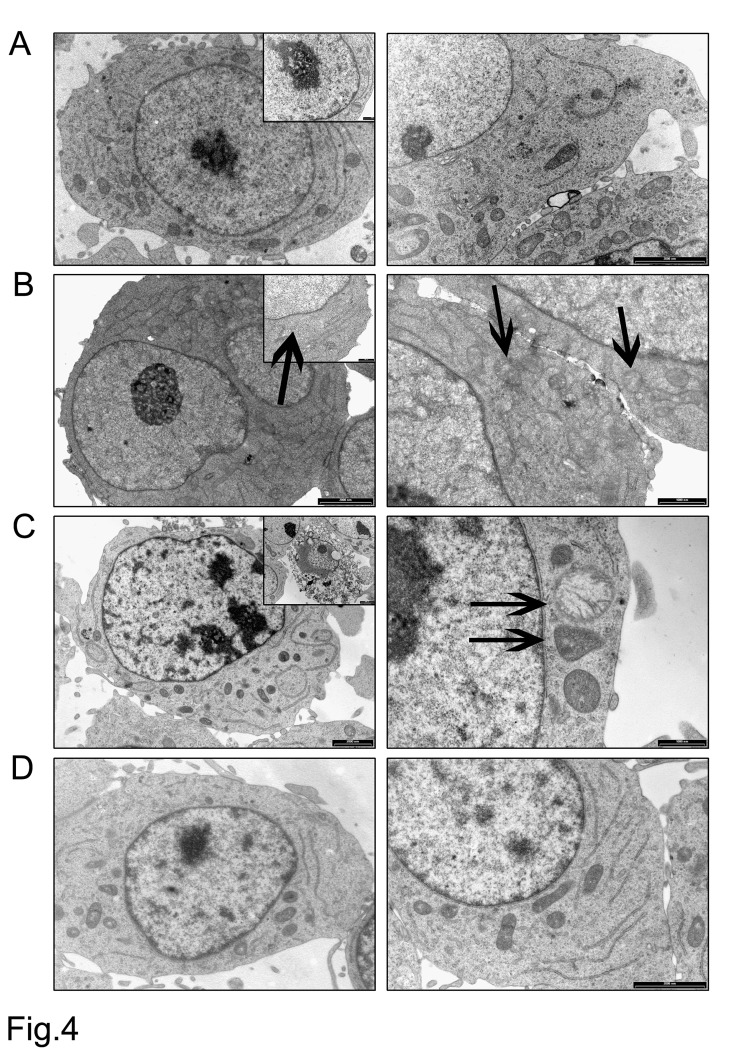
Electron microscopy analysis Ultrastructural features of EZN-2968 treated and untreated MM cells (A) MM1.S control cells; (B) EZN-2968 treated cells (20 μmol/L for 24h); (C) EZN-2968 treated cells (20 μmol/L for 72h). (D) Cells treated with scrambled oligonucleotide (20 μmol/L for 24h).

We investigated the ultrastructural changes induced by the HIF-1α antagonist on MM1.S by electron microscopy (EM). As shown in Fig. 4A, the MM1.S control cells showed the typical characteristic of B cells: a regular contour, a well-organized rough endoplasmic reticulum, regularly-shaped mitochondria of uniform density, and a large nucleus with diffusely and evenly spread chromatin. When incubated with 20 μmol/L EZN-2968 for 24h, some MM1.S cells exhibited a distorted contour, some mitochondrial damage (swelling and/or disrupted cristae, matrix density reduction) and/or clumping of the chromatin (Fig. 4B). These abnormalities were markedly discernible when cells were treated for 72h with the HIF inhibitor, as shown in Fig. 4C. Noticeably, no ultrastructural anomalies were detected in MM1.S cells incubated with scrambled oligonucleotide at the same time intervals, suggesting that the structural changes displayed by MM cells were directly connected with HIF-1α inhibition (Fig. 4D).

### HIF-1 suppression induces a mild apoptosis and cell cycle arrest

**Figure 5:**
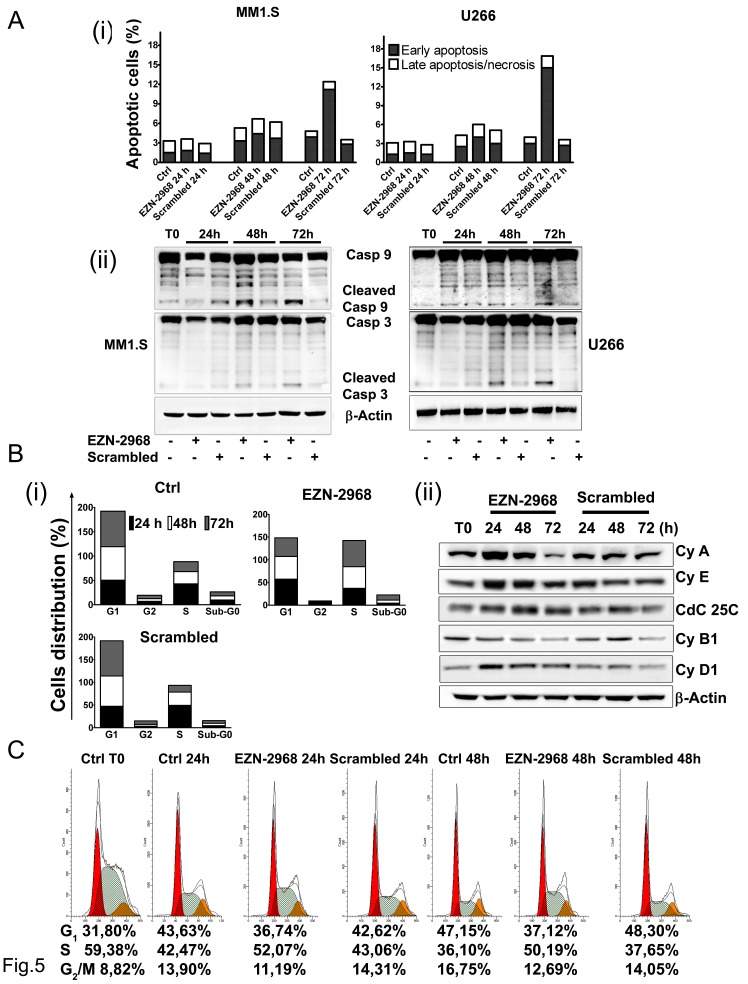
Effect of EZN-2968 on apoptosis and cell cycle on MM1.S (A) Early and late apoptotic events on MM1.S and U266 cells upon exposure to either EZN-2968 or scrambled oligonucleotide (20 μmol/L) for up to 72h were measured by flow cytometry. Staining for Annexin V and PI showed an increased fraction of Annexin V^+^PI^−^ events after 72h of incubation (i). Western blotting analysis of cleaved caspase 9 and 3 (ii). Similar results were obtained in two independent experiments. Representative data from one experiment are presented. (B) Cell cycle analysis was performed using PI staining. EZN-2968 blocked cell cycle progression in G_2_/M before inducing cell death at later time points (i). Analysis of proteins involved in cell cycle checkpoint control was carried out by western blotting. MM1.S cells were incubated with either EZN-2968 or scrambled oligonucleotide for up to 72h and whole cell lysates were then analyzed (ii). Representative data from one experiment are presented. (C) MM1.S cells were synchronized in S-phase using 2 mM thymidine for 48h and then released in complete media for additional 24h and 48h in the presence or absence of EZN-2968. Cell cycle profile was analyzed using propidium iodide (PI) staining (G_1_: *red*; S-phase: *grey*; G_2_/M; *orange*), and the corresponding percentages were reported under each histogram. Data were performed in two independent experiments with similar results. Representative data from one experiment are presented.

We next evaluated whether HIF-1α depletion could also affect the survival of MM cells. MM1.S cells were incubated for up to 72h with the HIF inhibitor and the percentage of cells undergoing apoptotic cell death was evaluated using AnnexinV/PI staining. After 72h of incubation with EZN-2968, a moderate percentage of cell population was positive for Annexin V (12% and 17% of apoptotic cells for MM1.S and U266 cell lines, respectively) compared to control and scrambled-treated cells (Fig. 5A (i)), suggesting that a small amount of cells proceed early toward apoptotic death, as also confirmed by caspases cleavage at late time points (Fig. 5A (ii)). When the cell cycle profile was analyzed, treatment with EZN-2968 induced a progressive accumulation of MM cells in S-phase with reduction of G1 and G_2_/M phase, compared to controls (Fig. 5B (i) and Supplementary S4). In order to determine the mechanisms responsible for EZN-2968-induced S-phase arrest, we investigated the expression of proteins involved in cell cycle checkpoints control. We treated MM cell lines with HIF-1α antagonist or scrambled oligonucleotide (20 μmol/L) for up to 72h and analyzed by western blot the whole cell lysate. The S-phase delay in EZN-treated samples was correlated with a marked increase of cyclin A, cyclin E and cyclin D1 levels as early as 24h compared to the scrambled group (Fig. 5B (ii)), suggesting that the cells were able to proceed through S-phase. Moreover, the expression of cyclin B1 was reduced when cells were treated with the HIF inhibitor, indicating that the cells were not longer able to enter the M phase. Because Cdc25C is induced during S-phase, we evaluated whether the Cdc25C signalling pathway is relevant in determining the EZN-induced S-phase cell cycle arrest. As shown in Fig. 5B (ii), the expression of Cdc25C was up-regulated in treated samples compared to the control, further supporting that HIF inhibition directly correlates with S-phase cell cycle delay. Interestingly, up-regulation of the cyclin A was observed either in p53 wild-type (MM1.S) and in p53 mutant (RPMI8226, and OPM-2) MM cell lines, suggesting a p53 independent regulation (data not shown). To confirm our results, we first treated MM1.S with thymidine (2 mM) to synchronize cells in S-phase and, after adequate wash out we cultured the cells in the presence of EZN-2968 for an additional 24h and 48h. Cell cycle profiles showed about 50% of cells in S-phase at each time point in EZN-2968-treated samples compared to controls (Fig. 5C). Similar results were obtained in U266, RPMI8226 and OPM-2 cell lines (data not shown). Taken together these data suggest that HIF-1α inhibition delays cell cycle progression by arresting cells in S-phase with only a minority of population proceeding to apoptotic death. The mechanism of HIF inhibition-induced S-phase arrest might involve both Cdc25C signalling pathway and cyclin A up-regulation.

### Biochemical phenotype of MM cells surviving EZN-2968 exposure

**Figure 6:**
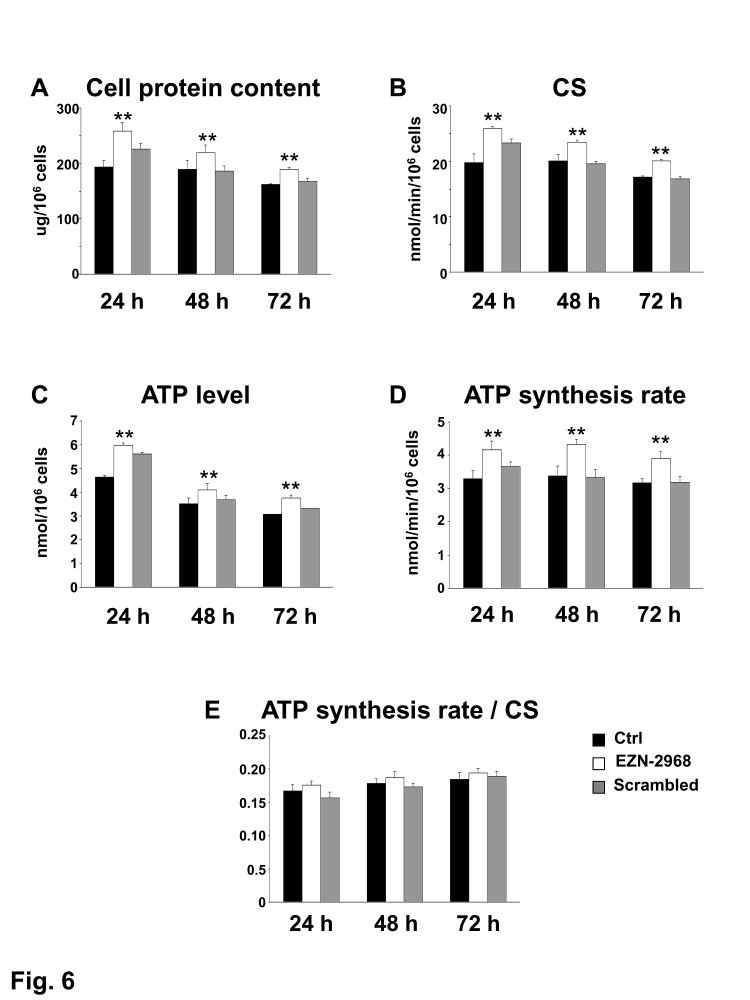
Biochemical changes in EZN-2968 treated MM cells The biochemical parameters were analyzed on viable MM1.S cells isolated from samples treated for up to 72h with either EZN-2968 or scrambled oligonucleotide by using the Cell Death Removal Kit. (A) The cellular protein content was expressed as μg protein/10^6^ cells. (B) The citrate synthase activity (CS), taken as an index of mitochondrial mass, was expressed as nmol/min/10^6^ cells. (C) The intracellular ATP content was measured as nmol/10^6^ cells. (D) The oligomycin-sensitive ATP synthesis rate (nmol/min/10^6^ cells) was measured in permeabilized cells energized with glutamate-malate. (E) To correct for mitochondrial mass, the OXPHOS rate measured in the presence of glutamate-malate was normalized to the citrate synthase activity. Data are presented as the mean value ± SD of three independent experiments. Statistical significance: **p<0.01 EZN-2968 treated vs control cells. EZN-2968-*white bars*, Scrambled-*grey bars*, Control-*black bars*.

**Figure 7:**
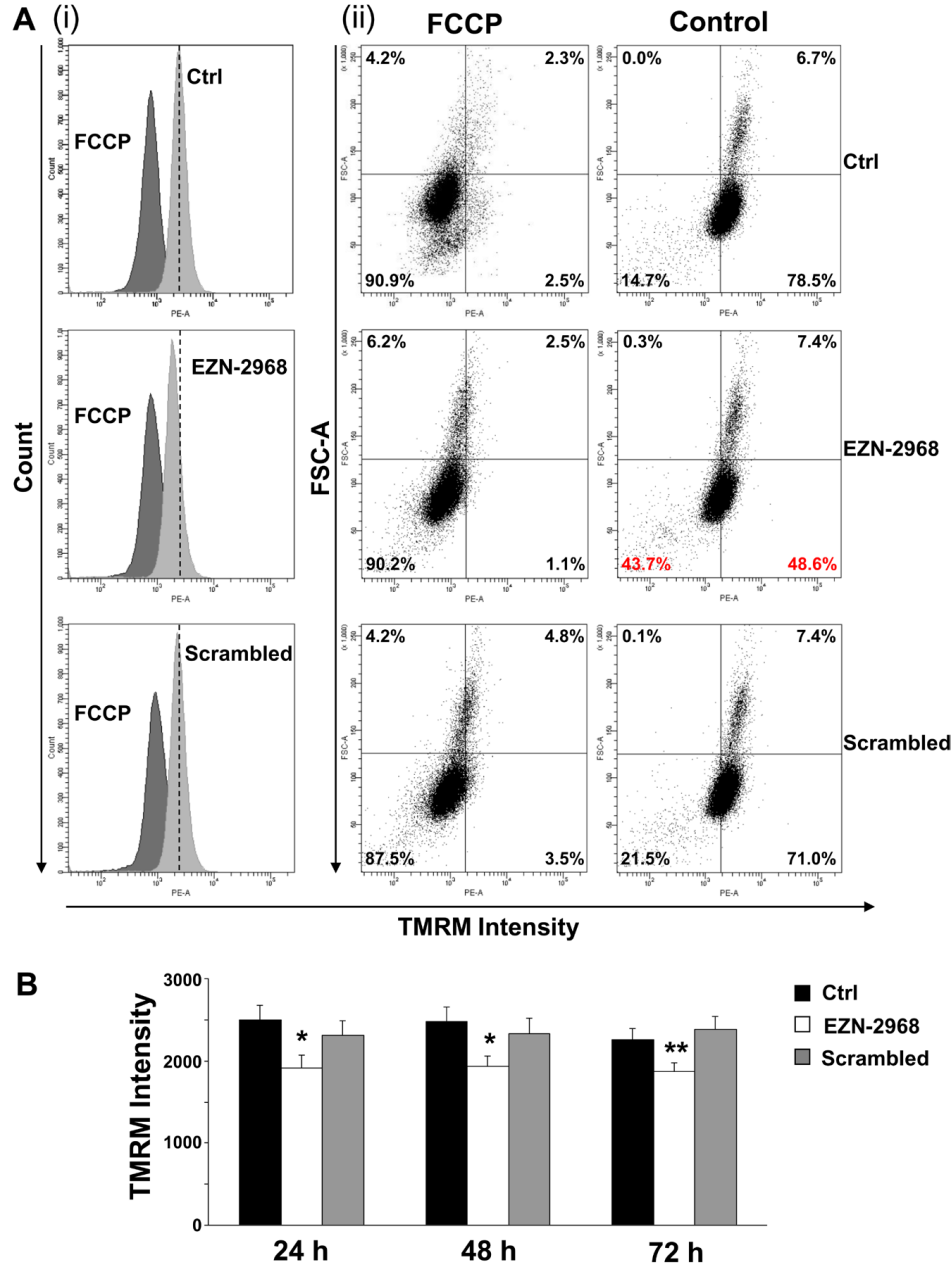
Mitochondrial membrane potential in EZN-2968 treated MM1.S cells Non-quenching mode analysis of mitochondrial membrane potential in viable intact cells loaded with 20 nM TMRM. (A) Representative graphs of mean fluorescence intensity (i) and distribution of percentages (ii) of MM1.S cells untreated or treated with either EZN-2968 or scrambled oligonucleotide for 24h. (B) TMRM intensity measured in MM1.S cells treated with HIF-1α inhibitor for up to 72h. Values shown in histogram are mean ± SD of three independent experiments. * p<0.05 and ** p<0.01 compared to control. EZN-2968-*white bars*, Scrambled-*grey bars*, Control-*black bars*.

Among the hallmarks of many cancer cells is the reduced mitochondrial function and increase of glycolysis to generate ATP even in the presence of normal oxygen tension, the so-called Warburg effect [39]. A number of studies have associated HIF-1 to this metabolic shift, suggesting us that EZN-2968 could modulate the mitochondrial function of myeloma cells by reducing the HIF-1α gene expression and HIF-1 level. The biochemical phenotype of MM cells was analyzed on viable cells obtained by removing both early and late apoptotic cells from EZN-2968/scrambled-treated or untreated samples. As shown in Fig. 6A, the EZN-2968 treatment of transformed cells results in significant increase of total cell protein content, being maximum at 24h and slightly lower at 48 and 72h. Similarly, the activity of citrate synthase (CS), a critical enzyme in the control of the mitochondrial flux through the trycarboxylic acid cycle, was found to increase upon exposure to EZN-2968, suggesting that the pharmacological treatment could also induce mitochondrial mass enhancement (Fig. 6B). This observation pushed us to evaluate the effect of EZN-2968 on the ATP cellular content and the mitochondrial ATP synthesis rate (i.e. Oligomycin-sensitive ATP synthase activity, OS-ATPase). Indeed, both ATP level (Fig. 6C) and OS-ATP synthesis rate (Fig. 6D) were significantly enhanced by EZN-2968 cell treatment, reaching a 25-30% increase at 24h of exposure. Since citrate synthase is an index of the cell mitochondrial mass [40], we normalized the OS-ATP synthesis rate to CS, resulting in a ratio that expresses the efficiency of the mitochondrial oxidative phosphorylation (OXPHOS) machinery. As shown in Fig. 6E the OXPHOS efficiency of MM cells was not altered by the pharmacological treatment at any time examined. Finally, the effect of EZN-2968 on the endogenous mitochondrial membrane potential (ΔΨ_mit_), a crucial parameter of mitochondrial function [38, 40] strictly bound to OXPHOS, was examined by cytofluorometric analysis of TMRM loaded cells. Fig. 7 shows that EZN-2968 treatment of MM cells decreased ΔΨ_mit_ as detected by both the TMRM mean fluorescence intensity (Fig. 7 (i)) and the distribution of the cell population (Fig. 7 (ii)). Notably, fluorescence intensity and distribution of TMRM loaded cell samples were evaluated with respect to the parameters obtained in the presence of FCCP, a protonophore capable to uncouple the mitochondrial respiration from ADP phosphorylation by dissipating the transmembrane proton gradient originated by respiration, therefore FCCP reduces ΔΨ_mit_ to its minimum. Moreover, ΔΨ_mit_ resulted significantly reduced at any time examined of the cells exposure to the HIF-1α inhibitor, as revealed by the decreased mean fluorescence intensity (Fig. 7B). This time-independent behaviour clearly indicates that EZN-2968-treated myeloma cells are more prone than controls to produce ATP via OXPHOS, contributing to the observed intracellular ATP level enhancement together with the observed mitochondrial mass increase.

## DISCUSSION

The major finding of the present study is the demonstration that HIF-1α suppression in MM cells reduces their viability by inducing cell cycle arrest in the S-phase and pushes cells towards a mitochondrial oxidative metabolism.

We have shown that established MM cell lines and primary CD138^+^ cells under normoxic conditions accumulate aberrant HIF-1α protein confirming previous data reported by Hu *et al.* [30]. In addition, we have shown that the locked nucleic acid antisense oligonucleotide EZN-2968 can reverse the HIF-1 dependent angiogenic and metabolic effects in MM cells. Notably, the effects of the oligonucleotide were obtained by simply incubating the molecule with the MM cells (i.e. avoiding transfection protocols).

Besides being induced by environmental hypoxia or growth factors, HIF-1α expression and stabilization in MM cells can arise from genetic abnormalities such as loss of tumor suppressor activity [12, 15]. Of note, in recent studies the c-MYC proto-oncogene, which is de-regulated in about 30–50% of patients with advanced MM and is associated with a poor prognosis [41], has been shown to promote either constitutive HIF-1α protein activation, or aberrant VEGF production by plasmacells (PCs) and angiogenesis under normoxic conditions [27, 41, 42]. In the present study, we have shown that inhibition of HIF-1α expression reduces VEGF release by MM cells, suggesting an anti-angiogenic activity of EZN-2968. Because angiogenesis is one of the most powerful drivers of MM progression, we believed of interest to characterize the effects of drugs capable to interfere with the HIF-1 induced pathways.

Interestingly, we observed that EZN-2968 causes cell cycle arrest in S-phase preventing the progression to mitosis. Progression through the eukaryotic cell cycle is controlled by the cyclin dependent kinases (Cdks) [43]. As a result of EZN-2968 exposure, we observed a modulation of important proteins involved in cell cycle checkpoints. Indeed, the expression of Cyclin E and Cyclin A were signiﬁcantly increased, whereas M-phase Cyclin B1 was markedly down-regulated. In particular, Cyclin E is a critical regulator of the G1/S transition, and its over-expression, after genotoxic stress, such as γ-irradiation, has been reported to induce apoptosis of hematopoietic cells through activation of the caspase cascade [44]. This pro-apoptotic effect was associated with an increased production of cyclin E/Cdk2 kinase activity [45]. Moreover, in agreement with previous studies on solid tumors [46], inhibition of HIF-1α gave rise to a progressive accumulation of cells in S-phase, while only a small percentage of cells entered in apoptotic phase. Therefore, our data suggest that EZN-2968-induced S-phase arrest resulted from enhanced initiation and progression of S-phase and concomitant inhibition of mitosis progression. Based on these observations, we speculate that HIF-1 inhibition first activates the Cyclin E/Cyclin A pathway, causing S-phase arrest, then it triggers caspases activation, eventually leading to apoptotic cell death after prolonged exposure, supporting the view that HIF-1α inhibition may be an attractive therapeutic strategy for MM.

Moreover, IGF-1, one of the most crucial growth factors for MM cells, was clearly shown to enhance HIF-1 levels, supporting that HIF-1α lies downstream of the IGF-1 signalling pathway in MM cells [30].

The benefit of EZN-2968 employment as a therapeutic molecule in MM clinical treatment was also supported by the biochemical phenotype of EZN-2968–treated MM cells. We showed that the HIF-1α pathway has major effects on MM cells and demonstrated that EZN-2968 treatment can reverse the Warburg cell phenotype pushing tumor cells towards a normal cell metabolism.

Approximately, 60% to 90% of cancers display the so-called Warburg phenotype, characterized by dependence on glycolysis as the major source of energy, irrespective of the oxygen level [47,48]. Due to the Warburg effect, pyruvate, the end product of glycolysis, is mainly converted into lactate by lactate dehydrogenase A (LDH-A) that is up-regulated in transformed cells. As recently reported, the Warburg effect is operative in MM cells, that are characterized by high expression levels of glycolytic enzymes and by the key regulators of the Warburg effect, LDHA and PDK1 [25]. In the present study, we have demonstrated that treatment with EZN-2968 (i) increases the mitochondrial mass, (ii) enhances the cell's ability to produce ATP via OXPHOS and (iii) that MM cells are forced to produce ATP via OXPHOS pushing the oxygen dependent oxidative metabolism as clearly shown by both the increased OXPHOS rate and the decrease of the endogenous mitochondrial membrane potential. Interestingly, the major structural and functional mitochondrial features of MM cells match the main characteristics of human fibroblasts adapted to hypoxia as it has been recently reported by Baracca *et al.* [49], confirming the central role of HIF-1 in the control of cell metabolism. One of the novelty of the present study is that the suppression of HIF-1α in MM cells induces morphological and functional changes leading to reversal of the Warburg effect, that parallels the return of fibroblasts to the normoxic metabolism [49].

Despite availability of several therapeutic agents, MM still remains an incurable disease. Thus, the development of new therapies are urgently needed. Because elevated expression of HIF-1α protein has been correlated with drug resistance and overall poor treatment outcome, compounds that inhibit HIF-1α may have broad utility in the control of cancer growth [23]. Although many small molecules affect HIF-1α levels indirectly [23], none of them specifically inhibits HIF-1α. In this study we used a novel and highly specific antagonist of HIF-1α mRNA (EZN-2968), which potently and selectively reduces HIF-1α mRNA and protein levels. While several criticisms have been raised concerning the use of oligonucleotides because of their poor delivery into the cells, herein we clearly demonstrate that EZN-2968 has a high delivery efficiency into MM cells avoiding any transfection protocols. This intrinsic feature of EZN-2968 increases its possible employment as a therapeutic molecule in clinical practice. Moreover, the present study discloses novel mechanisms that support the anticancer action of EZN-2968 adding further interest to its specificity and well tolerance as revealed in Phase I studies. Furthermore, agents that inhibit HIF-1α may be particularly well suited for combination therapy with other anticancer agents. In conclusion, our study highlighted the role of HIF-1α as a critical regulator of MM cells, providing evidence that PCs homeostasis is sustained by HIF-1α and therefore HIF inhibition can represent a main target for MM therapy.

## MATERIALS AND METHODS

### Compounds

EZN-2968 (anti-HIF-1α) and EZN-3088 (negative control or Scrambled) were obtained from Enzon Pharmaceuticals Inc. (Piscataway, New Jersey) [35]. Recombinant human interleukin 6 (IL-6) and insulin like growth factor 1 (IGF-1) were purchased from R&D System (Minneapolis, MN, USA), whereas phytohemagglutinin (PHA-P) and cobalt chloride (CoCl_2_) from Sigma-Aldrich (St. Louis, MO, USA).

### Cell culture and cells synchronization

Human MM cell lines U266, RPMI8226 and OPM-2 were purchased from DMSZ Germany, whereas MM1.S cells were kindly provided by Dr. Renate Burger (Division of Stem Cell Transplantation and Immunotherapy, Kiel, Germany) and cultured in RPMI-1640 media (BioWhittaker, Walkersville, MD, USA) supplemented with 10% of FBS (BIOCHROMAG, Leonornstr., Berlin, Germany), 2 mM L-Glutamine (BIOCHROMAG, Leonornstr., Berlin, Germany), streptomycin 100 U/ml, penicillin 100 U/ml and maintained in 5% CO_2_ at 37°C. Hypoxia (1% pO_2_) was generated in an Vivo2300 hypoxic workstation (Ruskinn Technologies, Ireland). Mononuclear cells from bone marrow aspirates or peripheral blood samples were isolated by density gradient centrifugation over Ficoll-Paque Plus (Amersham Biosciences, Piscataway, NJ, USA). CD138^+^ and CD34^+^ cells were then isolated by immunomagnetic bead positive selection in a Mini MACS LS column following the manufacturer's protocol (Milteny Biotech, Auburn, CA). The purity of MM cells was confirmed by flow cytometry analysis using phycoerytrin-conjugated anti CD138/CD34 antibody (Milteny Biotech, Auburn, CA). Patient samples were collected after informed consent.

To analyze S-phase progression MM cells were synchronized in the presence of 2 mM Thymidine (Sigma Aldrich, St Louis, MO, USA) for 48h, then released in complete media for up to 48h. Cell cycle analysis was then performed.

### RNA isolation and Quantitative real-time PCR

Total RNA was isolated using RNeasy mini kit (Qiagen, Valencia, CA) with an automated RNA extraction method according to the manufacturer's instructions (QIAcube, Qiagen, Valencia, CA). 100 ng of total RNA was reverse transcribed using SuperScript™ III First-Strand Synthesis System and random hexamers (Invitrogen Life Technologies). Real-time PCR (qRT-PCR) was performed by adding 2 μl of 20 μl cDNA to an universal master Mix (Lightcycler probe Master mix, Roche, Applied science), primers (0.5 μmol/L of each primer) and universal probes, UPL (0.2 μmol/L of each probe). UPL probe #66, #60 and #17 were used to quantified HIF-1α, GAPDH and HIF-2α respectively. The following primers pairs were used: HIF-1α forward 5'-tttttcaagcagtaggaattgga-3' and reverse 5'-gtgatgtagtagctgcatgatcg-3'; HIF-2α forward 5'-gacatgaagttcacctactgtgatg-3' and reverse 5'-gcgcatggtagaattcatagg-3'; GAPDH forward 5'-agccacatcgctcagacac-3' and reverse 5'-gcccaatacgaccaaatcc-3'. All reactions were performed in triplicate using the LightCycler® 480 instrument (Roche, Applied science) in a total volume of 20 μl.

### Western blotting and Co-immunoprecipitation (Co-IP)

For western blotting and Co-IP analyses, cells pellets were lysed by using modified RIPA buffer (5 mM EDTA, 2 mM Na_3_VO_4_, 5 mM NaF and 1 mM PMSF) supplemented with protease inhibitor cocktail (Sigma-Aldrich, St Louis, MO, USA). For western blotting analysis, SDS-PAGE was performed, and proteins were electroblotted onto PVDF membranes. Co-IP analyses were performed using 300 μg proteins from whole cells lysates. Samples were incubated overnight in IP buffer (250 mM NaCl, 15 mM MgCl_2_, 40 mM Hepes, 60 mM glycerophosphate) additioned with protease inhibitors with primary antibody in presence of CNRr-activated sepharose 4B (Pharmacia), resolved by SDS-PAGE, transferred into PVDF membranes and labeled with anti-HIF-1α and/or anti-p300 primary antibodies. The primary antibodies used for Immunoblotting were purchased from: Novus Biologicals, (anti-HIF-1α), Millipore™ (anti-p300 and anti-Cyclin A), Santa Cruz (anti-Cdc25C, anti-Cyclin E and anti-β-Actin), Cell Signaling Technology® Inc (anti-Cyclin B1, anti-Cyclin D1, anti-Caspase 3 and anti-Caspase 9) and Oncogene Research (anti-p53). The secondary antibodies used for Immunoblotting were purchased from: GE Healthcare (anti-rabbit and anti-mouse) and Santa Cruz (anti-goat).

### Immunofluorescence Microscopy analysis

Cells set on poly-L-lysine-coated glass slides for 3h were fixed and permeabilized following standard protocols, and then incubated overnight at 4°C with primary HIF-1α antibody (1:1000 in 1% BSA in PBS). Incubation with secondary antibody (anti-rabbit conjugated with FITC from Santa Cruz Biotechnology 1:2000) was performed for 2h at room temperature followed by 15 min incubation with DAPI (1:100 dilution in PBS). Images were obtained using an Axiovert 40 CFL microscope (Carl Zeiss MicroImaging GmbH, Germany) using 100X objective, and analyzed using the AxioVision 4.7 imaging system software.

### ELISA Assay

VEGF levels secreted by MM cells treated with EZN-2968 or scrambled oligonucleotide (20 μmol/L) were quantified using Human VEGF Quantikine® kit (R&D Systems, Minneapolis, MN, USA) according to the manufacturer's instructions. Cell cultures media were harvested for up to 96h. All standards and samples were tested in triplicate.

### Cell viability assessment

Cell viability was measured by MTT colorimetric survival assay (Sigma-Aldrich, St. Louis, MO, USA). In brief, cells were plated in 96 well plates at density of 50.000 cells/well. EZN-2968/EZN-3088 were added at the concentration indicated and compared with the controls. Cultures were then incubated for up to 96h in a 37°C incubator with 5% of CO_2_. Optical absorbance of the culture medium was then measured using a multiplate reader (Multiskan Ex Microplate Photometer, Thermo Scientific, Meridian Rd, Rockford, USA). Each experiment was done in triplicate.

### Cell death commitment assay

The minimum exposure of MM cells to EZN-2968 required to commit them to death was evaluated by incubating cells in well plates with EZN-2968 (20 μmol/L) for up to 96h. Following incubation, the cells were washed twice to remove any residual drug, and then incubated in drug-free medium for up to 72h. MM cell survival was quantified by MTT survival assay and expressed as percentage of the respective controls value.

### Electron microscopy

The ultrastructural features of treated and control MM1.S cells were investigated by transmission electron microscope (TEM). To maintain the natural morphology, cells were rapidly washed and fixed in Karnowsky fixative (2% glutaraldehyde, 4% formaldehyde in 0.1M phosphate buffer) directly in culture plate for 20 min at room temperature (RT). After mechanical removal with scraper, the cells were pelleted, fixed again with the same fixative for 24h at 4°C and processed for TEM analysis. After a post-fixation in 1% buffered osmium tetroxide for 1h at RT, samples were dehydrated through graded ethanol and embedded in Araldite resin. Serial semi-thin sections were obtained with an ultramicrotome and stained with Toluidine blue. Grids containing ultrathin sections were contrasted with uranyl acetate and lead citrate before observing with a Philips 400T (FEI Company, Milan, Italy) transmission electron microscope.

### Annexin V staining and cell cycle analysis

Cellular apoptosis was evaluated using FITC Annexin V Apoptosis Detection Kit I (BD Pharmingen™), according to the manufacturer's instructions. Cell fluorescence and PI uptake were measured by mean of a FACScan flow cytometer and a dedicated software (FACS Canto2, BD Pharmingen™). Cell cycle analysis was evaluated using the PI/RNase Staining Buffer (BD Pharmingen™), according to the manufacturer's instructions. PI uptake was measured by mean of a FACScan flow cytometer and analyzed with ModFitLT version 3.1 (Verity software).

### Biochemical assays

The Biochemical phenotype of EZN-2968 treated MM cells was analyzed on viable cell samples using a Cell Death Removal Kit (Miltenyi Biotec Inc.).

Protein content was determined by the method of Lowry [36] and the viable cells were counted by means of the trypan blue exclusion test. The cellular protein content was expressed as μg of protein/10^6^ cells. Citrate synthase activity was assayed as previously reported [37]. Essentially, cells were incubated with 0.02% Triton X-100 and the reaction was monitored by measuring spectrophotometrically the rate of free coenzyme A released. The enzyme activity was expressed as nmol/min/10^6^ cells. The oligomycin-sensitive ATP synthase activity was measured in permeabilized myeloma cells according to the luminometric method described by Sgarbi *et al* [37]. Essentially, cells (5 x 10^6^ cells/ml) were permeabilized with 60 μg/ml digitonin in a Tris/Cl buffer (pH 7.4). The reaction was started by adding 10 mM glutamate/malate (+ 0.6 mM malonate) and 0.5 mM ADP (all reagents were from Sigma-Aldrich, St. Louis, MO, USA). After 3 min of incubation, the reaction was stopped by adding dimethylsulphoxide and synthesised ATP was measured using a luciferin–luciferase system (ATP bioluminescent assay kit CLS II; Roche, Basel, Switzerland) according to the manufacturer's instructions. The rate of ATP synthesis was expressed as nmol/min/10^6^ cells. The total content of cellular ATP was assayed on dimethylsulphoxide extract from the cells sample using the luminometric assay reported above and it was expressed as nmol/10^6^ cells.

### Flow cytometry analysis of mitochondrial membrane potential

Mitochondrial membrane potential (ΔΨ_mit_) was measured by staining the cells in a non-quenching mode [38]. Cells were loaded with 20 nM tetramethylrhodamine methyl ester (TMRM) (Molecular Probes, Eugene, OR, USA) which accumulates in the mitochondrial matrix in a ΔΨ_mit_-dependent manner. The myeloma cells were incubated with the dye solution for 30 min at 37°C and then washed with PBS to remove any remaining unincorporated dye. The pellet was resuspended in HBSS and immediately analyzed with a FACScan flow cytometer (FACS Canto2, BD Pharmingen™). Excitation was at 488/10 nm and fluorescence emission was measured at 585/42 nm. Data acquisition and analysis was performed by BD DIVA 2 software.

### Statistical analysis

Data were analyzed by means of the two-way analysis of variance (ANOVA) with Bonferroni's post-hoc test. Statistical analysis was performed by running the GraphPad Prism® 4.0 software (GraphPad Software, Inc.) statistical package. Data are reported as mean values ± SD. Two-tailed P values of less than 0.05 were regarded as statistically significant.

## SUPPLEMENTARY FIGURES


