# Supplementary material for: Hypoxia inducible factor-1 alpha as a therapeutic target in multiple myeloma
Source: Oncotarget. 2014 Jan 23;5(7):1779–92. doi: 10.18632/oncotarget.1736 (PMC4039126; doi:10.18632/oncotarget.1736)
Supplement: Supplementary file 1 [file oncotarget-05-1779-s001.pdf]

# Hypoxia Inducible Factor-1 alpha as a therapeutic target in Multiple Myeloma – Borsi et al

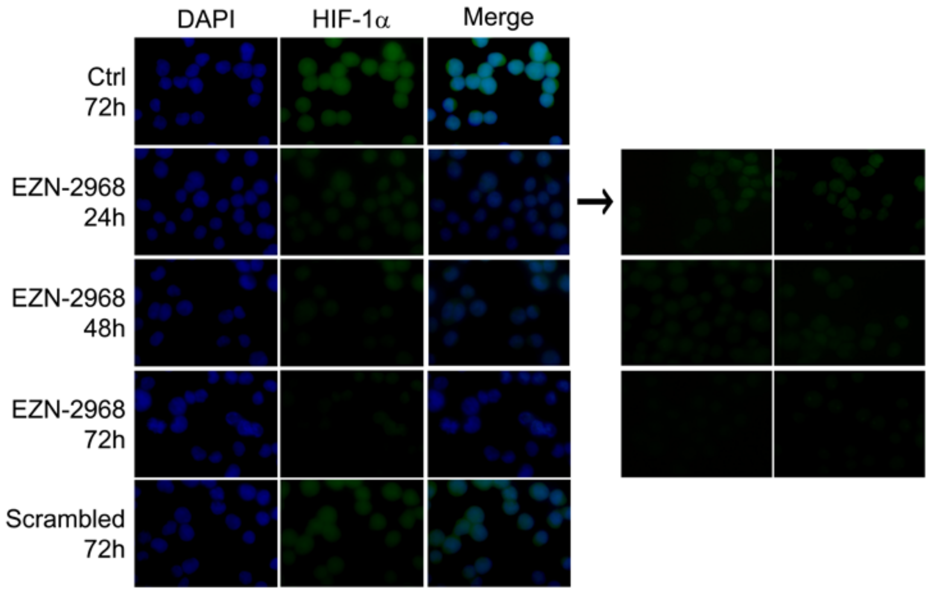


**Supplementary S1:** Immunofluorescence staining of HIF-1α in MM1.S cells treated with or without EZN-2968 (20 µmol/L) for up to 72h. For each experiment between 150 and 200 cells were evaluated per slide. The down-regulation of HIF-1α protein level was observed in a time dependent manner in EZN-treated samples compared to controls. Remarkably, EZN-2968 was able to deliver into MM cells homogeneously without using transfection methods. Green fluorescence: HIF-1α; Blue fluorescence: DAPI.

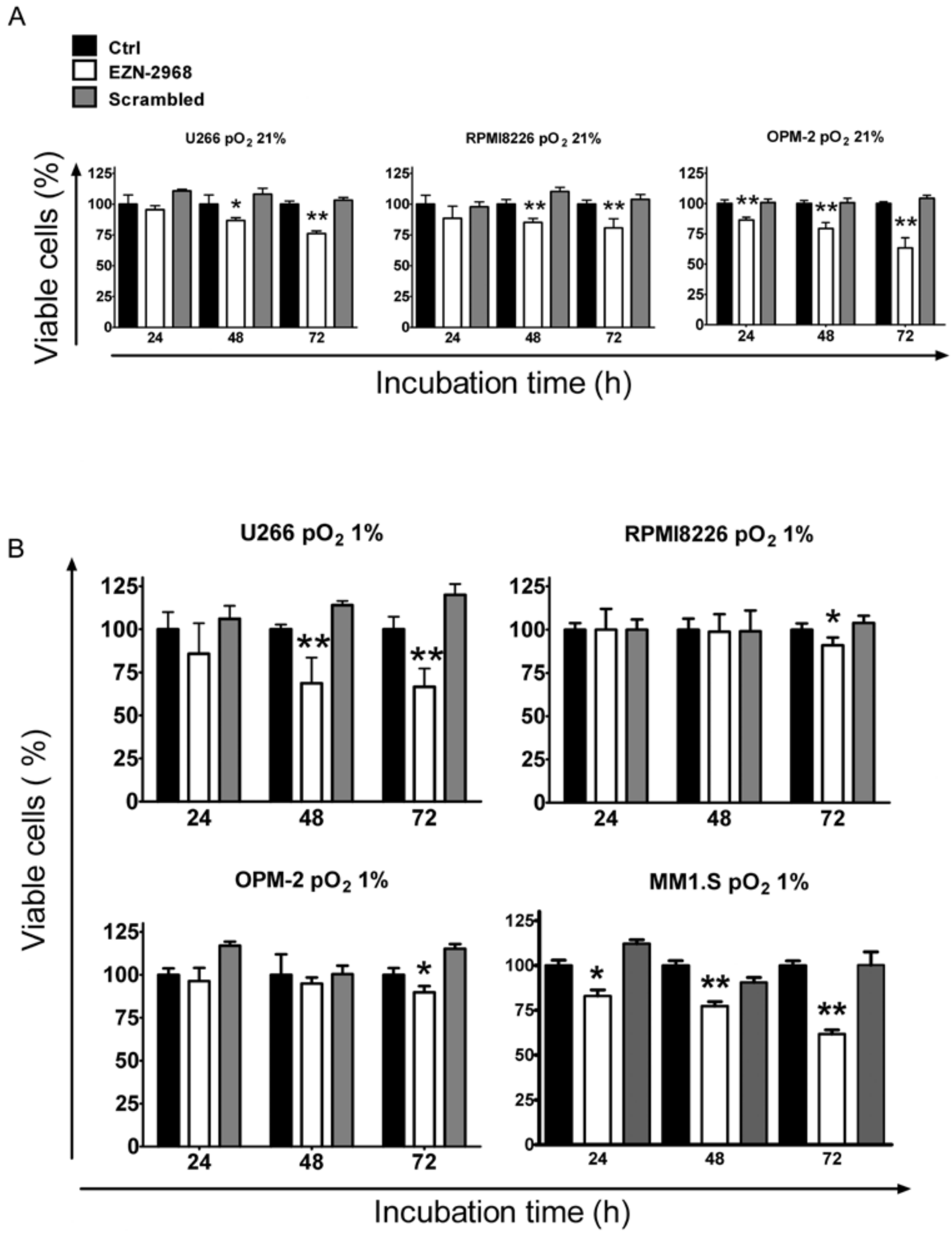


**Supplementary S2:** The viability of MM cell lines under normoxic ($pO_2$ 21%: U266, RPMI8226, OPM-2) (A) and hypoxic ($pO_2$ 1%: U266, RPMI8226, OPM-2 and MM1.S) (B) culture conditions was evaluated at 24h, 48h and 72h using the MTT colorimetric survival assay. Histograms show the mean value ± SD of three independent experiments. * $p<0.05$ and ** $p<0.01$ compared to control.

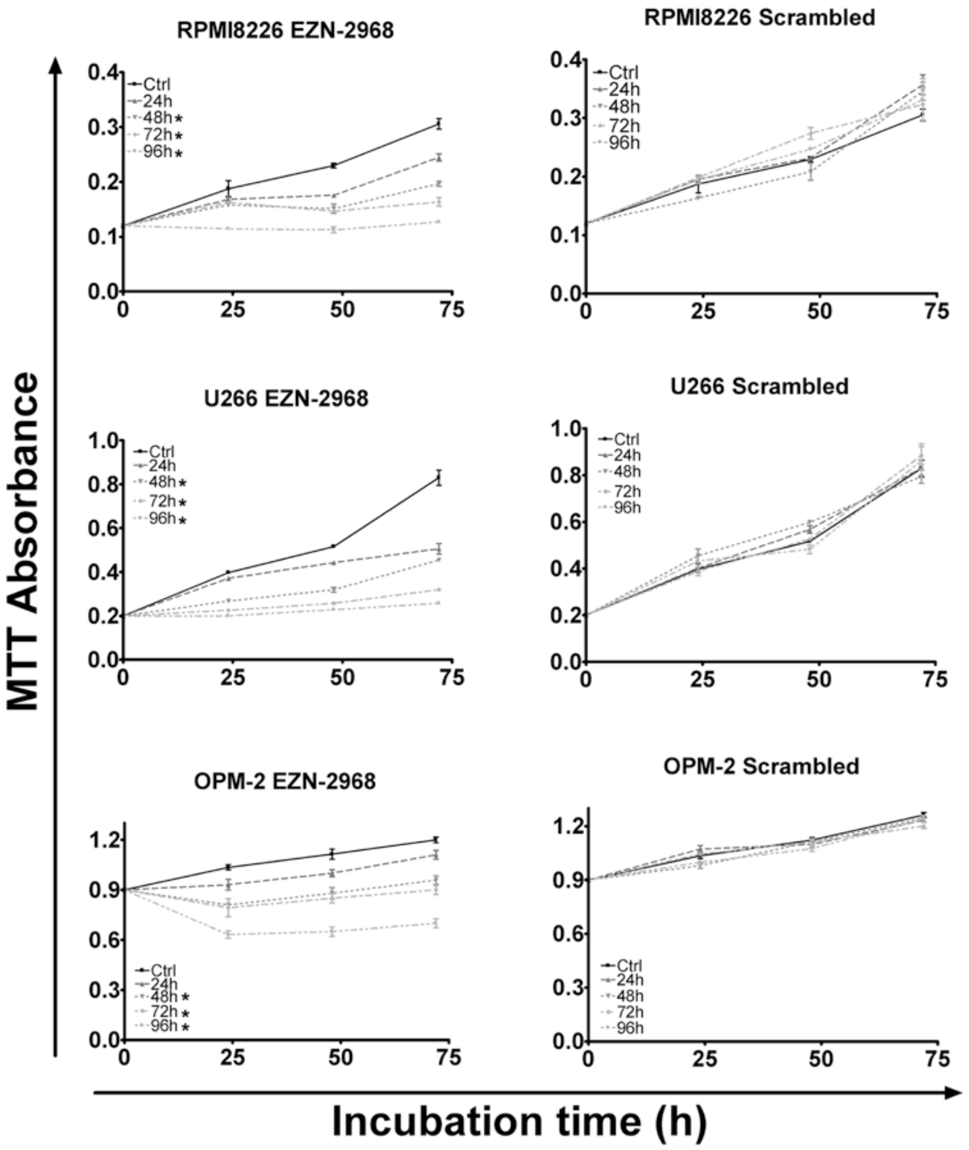


**Supplementary S3:** The minimum exposure to EZN-2968 required to commit MM cell lines to death was evaluated by incubating cells with 20 µmol/L EZN-2968 or scrambled oligonucleotide for 24-96h. Following incubation, the cells were washed and grown in drug-free medium for additional 3 days. MM cell survival was quantified by MTT colorimetric survival assay. Values shown are mean ± SD of three independent experiments. The data represented were statistically significant after 48h (* $p<0.05$ compared to control).

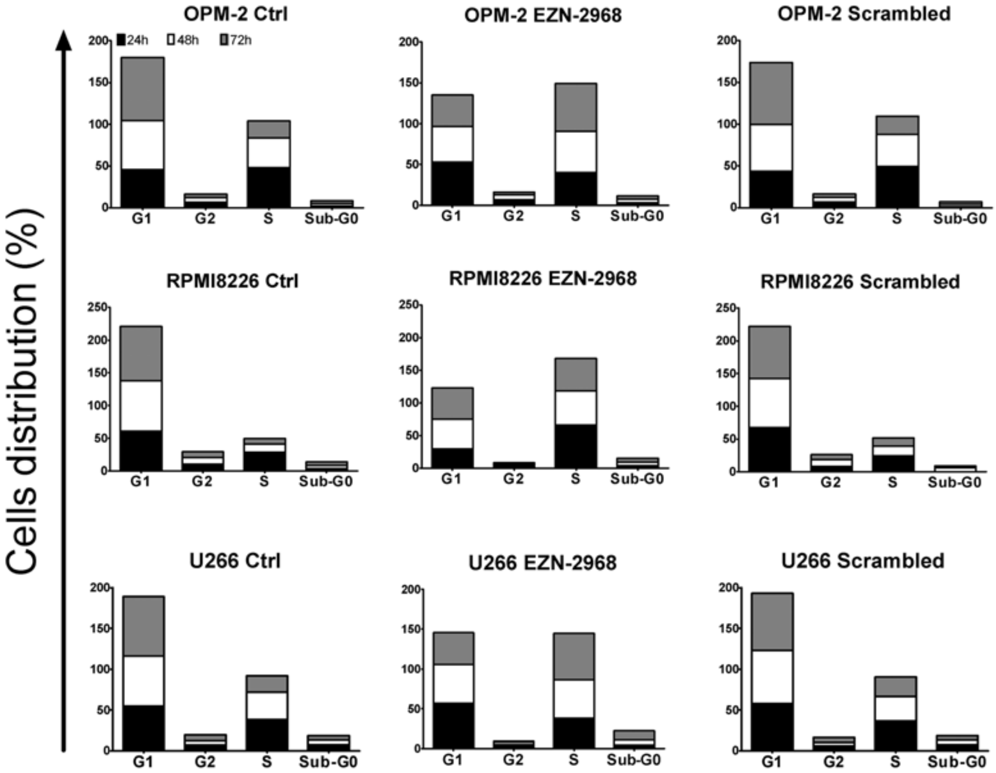


**Supplementary S4:** Cell cycle profile of MM cell lines (U266, RPMI8226, OPM-2) were analyzed using propidium iodide (PI) staining. MM cells were incubated in the presence of EZN-2968 or scrambled oligonucleotide for up to 72h. Data are representative of 2 independent experiments with similar results.
